# Humoral response after BNT162b2 vaccine in Japanese hemodialysis patients

**DOI:** 10.1186/s41100-022-00452-1

**Published:** 2023-02-20

**Authors:** Ryoichi Miyazaki, Kyoko Miyagi, Misaki Yoshida, Yasunori Suzuki

**Affiliations:** Department of Internal Medicine, Fujita Memorial Hospital, 4-15-7, Fukui, Fukui, 910-00004 Japan

**Keywords:** COVID-19 prevention, BNT162b2 vaccine, Japanese hemodialysis patients

## Abstract

**Background:**

Hemodialysis patients are more likely to be severely affected if infected by COVID-19. Contributing factors include chronic kidney disease, old age, hypertension, type 2 diabetes, heart disease, and cerebrovascular disease. Therefore, action against COVID-19 for hemodialysis patients is an urgent issue. Vaccines are effective in preventing COVID 19 infection. In hemodialysis patients, however, responses to hepatitis B and influenza vaccines are reportedly weak. The BNT162b2 vaccine has shown an efficacy rate of about 95% in the general population, but as far as we know there are only several reports of efficacy data in hemodialysis patients in Japan.

**Methods:**

We assessed serum anti-SARS-CoV-2 IgG antibody (Abbott SARS-CoV-2 IgG II Quan) in 185 hemodialysis patients and 109 health care workers. The exclusion criterion was positivity for SARS-CoV-2 IgG antibody before vaccination. Adverse reactions to BNT162b2 vaccine were evaluated through interviews.

**Results:**

Following vaccination, 97.6% of the hemodialysis group and 100% of the control group were positive for the anti-spike antibody. The median level of anti-spike antibody was 2,728.7 AU/mL (IQR, 1,024.2–7,688.2 AU/mL) in the hemodialysis group and 10,500 AU/ml (IQR, 9,346.1–2,4500 AU/mL) in the health care workers group. The factors involved in the low response to the BNT152b2 vaccine included old age, low BMI, low Cr index, low nPCR, low GNRI, low lymphocyte count, steroid administration, and complications related to blood disorders.

**Conclusions:**

Humoral responses to BNT162b2 vaccine in hemodialysis patients are weaker than in a healthy control sample. Booster vaccination is necessary for hemodialysis patients, especially those showing a weak or non-response to the two-dose BNT162b2 vaccine.

*Trial registration* UMIN, UMIN000047032. Registered 28 February 2022, https://center6.umin.ac.jp/cgi-bin/ctr/ctr_reg_rec.cgi.

## Background

The World Health Organization reported that as of October 5, 2022, there have been over 615 million confirmed cases of COVID-19 worldwide and over 6.5 million deaths from the disease [1]. Moreover, hemodialysis patients contracting COVID-19 are reportedly and increased risk of severe disease and mortality because they are often immunocompromised [2]. Vaccines have been shown to effectively combat COVID-19 in the general population [3]; however, COVID-19 vaccines appear to be less effective in hemodialysis patients [4–16]. Factors associated with the weak response to the COVID-19 vaccine in hemodialysis patients include older age [8, 13–16], low serum albumin, low lymphocyte counts, high intravenous iron dosage, and high body mass index (BMI), though there are differences among the reported findings [4–7, 13–16].

Adverse events associated with the BNT162b vaccine include fever, general malaise, headache, chills, myalgia, arthralgia, and pain at the site of vaccination [3]. However, many reports indicate that adverse events after vaccination are milder in hemodialysis patients than in the general population [17, 18]. There are few reports in English on the results of COVID-19 vaccination in Japanese hemodialysis patients. Our aim was to assess the humoral response following vaccination with the BNT162b2 vaccine in patients on maintenance hemodialysis and the factors associated with those responses.

## Methods

### Study design and setting

This observational, prospective, single-center study to evaluate the humoral response after BNT162b2 (Pfizer-BioNTech) vaccination in Japanese hemodialysis patients was carried out at Fujita Memorial Hospital. The BNT162b2 vaccine was administered in our dialysis facility. Vaccination (two doses, 30 μg each) took place between April 4, and May 22, 2021. As recommended by the manufacturer, there was a 21-day interval between the first and second doses. Follow-up continued until February 28, 2022.

### Participants

Overall, 185 hemodialysis patients (HD group) and a control group composed of 109 health care workers (HCW group) without kidney failure from our hospital were included in the study. The HCW group was our medical staff, whose eGFR was 76.2 ± 8.3 mL/min/1.73 m^2^. All hemodialysis patients over 18 years of age in our dialysis facility were considered for inclusion. Exclusion criteria for participants were: vaccination refusal (only one hemodialysis patient and two health care staff), a history of SARS-CoV-2 infection prior to vaccination, and positivity for anti-S IgG antibodies (> 50 AU/mL) prior to vaccination. The eGFR of the HCW group was 76.2 ± 8.3 mL/min/1.73 m^2^. The characteristics of the study participants are detailed in Table 1.

**Table 1:** Participant profiles

	HD (*n* = 185)	HCWs (*n* = 109)	*P*
Age	68.7 ± 12.0	50.6 ± 12.0	< 0.001
Male/Female	125/60	22/87	< 0.001
BMI kg/m^2^	22.2 ± 4.0	21.7 ± 3.0	0.553
eGFR ml/min/1.73m^2^		76.2 ± 8.3	
Dialysis vintage, months	106 ± 106	0	
DM/NDM	72/113	7/102	< 0.01
Hematological disorder (Yes/No)	8/177	0/109	0.028
Steroid(Yes/No)	16/169	1/108	0.004
Immunosuppressant (Yes/No)	4/181	0/109	0.301

*HD* hemodialysis patients; *HCWs* healthcare workers; *BMI* body mass index; *DM* diabetes mellitus; and *NDM* non-diabetes mellitus

### Humoral response assessment

Post-vaccination antibodies were assayed using a chemiluminescent microparticle immunoassay (SARS-CoV-2 IgG II Quant assay on an ARCHITECT analyzer; Abbott) to quantify IgG antibodies in the patient’s plasma. The assay detects antibodies against the receptor binding domain of the S1 subunit of the spike protein of SARS-CoV-2 and presents a positive predictive agreement of 99.4% (95% confidence interval [95% CI], 96.50% to 99.97%) and a negative predictive agreement of 99.6% (95% CI, 99.15% to 99.37%), and its results are consistent with those obtained using a neutralization method (positive agreement, 100.0%; 95% CI, 95.72% to 100.00%) [14, 15]. A value 50 arbitrary units per milliliter (AU/ml) was considered evidence of a vaccination response [19]. Anti-SARS-CoV-2-spike IgG antibody assays have shown excellent correlation with neutralizing antibodies [20]. Antibodies were measured before vaccination and at a median of 17 (IQR: 16–19) days after the second vaccination in both groups.

### Other variables

The Kt/V, PCR, nPCR, Cr index, and GNRI in the patients were calculated as described previously [21–23]. BMI was defined as dry weight in kilograms divided by height squared in meters. We used recorded laboratory tests, which were routinely conducted for all patients on hemodialysis at the beginning of the month prior to their first dose of the SARS-CoV-2 vaccine. Details on the patients’ maintenance were obtained from their medical charts.

### Adverse events

Using a standardized questionnaire, vaccination-related adverse events were separately assessed in the HD and HCW groups. They included fever, malaise, headache, chills, vomiting, diarrhea, myalgia, and injection site pain. We questioned HD patients and HCWs about the subjective severity of the adverse events after vaccine administration. The grading was divided into three levels: mild, moderate, and severe. The grades were established according to the Food and Drug Administration toxicity grading scale [24].

### Statistical analyses

All data for continuous variables are summarized and displayed as the mean (SD) in each group. For categorical variables, the chi-square statistic was used to assess the statistical significance between groups. Parameters were compared using t tests if normally distributed or Kruskal–Wallis/Mann–Whitney U tests if not normally distributed. *P* < 0.05 was considered statistically significant for all analyses. EZR Statistics for Windows, R version 3.4.1 (The R Foundation for Statistical Computing, Japan), was used for all statistical analyses [25].

## Results

### Participants

The HD group was significantly older, more male-dominated, had a higher rate of diabetes and hematologic complications, and received more steroids than the HCW group.

Humoral responses were assessed in 185 patients in the HD group and 109 healthy individuals in the HCW group, all of whom received two doses of BNT162b2 vaccine. There were no cases of dropout in either group during follow-up.

### Humoral response

**Fig. 1:**
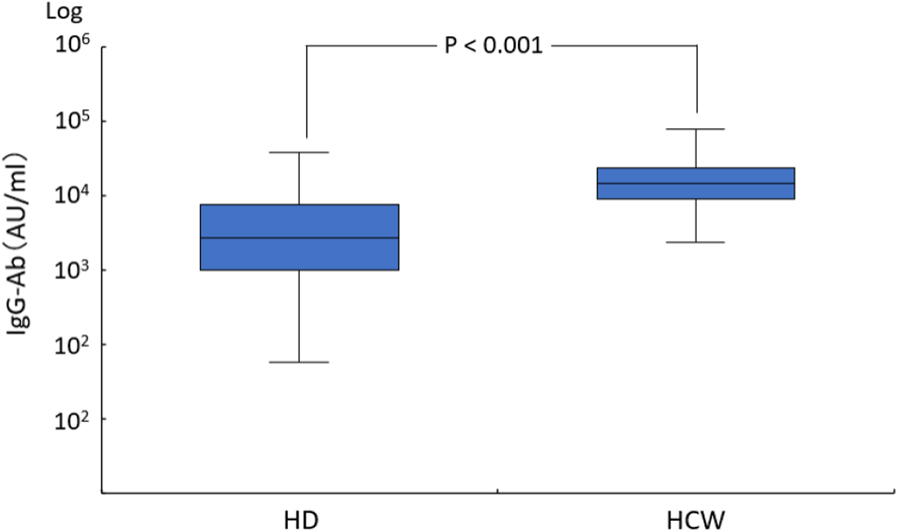
SARS-CoV-2 IgG antibody levels in the HD and HCW groups. The top and bottom borders of the boxes are the upper and lower quartiles, respectively. The medians are marked by horizontal lines inside the boxes. Error bars depict the range between minimal and maximal points. The antibody IgG level in the HD group (median 2,728.7, interquartile range 1,024.2-7,688.2) was significantly lower than in the HCW group (median 10,500, interquartile range 9,346.1-24,500)

The anti-SARS-CoV-2 IgG antibody positivity rate after BNT162b2 vaccine administration was 181/185 = 97.8% in the HD group and 109/109 = 100% in the HCW group. However, the IgG level in the HD group (median 2,728.7, interquartile range 1,024.2–7,688.2) was significantly lower than in the HCW group (median 10,500, interquartile range 9,346.1–24,500 (Fig. 1). The HD group was significantly older than the HCW group (Table 1), but even after corrected for age, the anti-SARS-CoV-2-spike IgG antibody level was significantly lower in the HD than the HCW group (data not shown).

### Factors associated with a weak humoral response after vaccination

The factors associated with the lower vaccine responsiveness in the HD group were older age, lower lymphocyte count, lower nPCR, lower GNRI, lower Cr index, lower BMI, current smoking, steroid administration, and hematological complications (Table 2). Comparison of the quartile with the lowest of antibody titer (LQ) to the quartile with the highest titer (HQ) showed that the mean age was 72.0 ± 10.2 years in the LQ group and 65.4 ± 13.3 years in the HQ group, a difference of about 7 years. The lymphocyte count was 940 ± 340/mm3 in the LQ group and 1,219 ± 436/mm3 in the HQ group. The nPCR was 0.84 ± 0.19 g/day/kg in the LQ group, which was significantly lower than the 0.94 ± 0.250.19 g/day/kg in the HQ group. The PCR, on the other hand, did not differ between the two groups. The GNRI was 91.7 ± 5.0 in the LQ group and 94.7 ± 5.1 in the HQ group. The Cr index and BMI were, respectively, 86.2 ± 26.9 and 21.2 ± 3.8 kg/m2 in the LQ group and 99.9 ± 28.0 and 22.9 ± 4.6 kg/m2 in the HQ group. The number of patients taking oral steroids was 9 out of 46 in the LQ group and 1 out of 46 in the HQ group. There were 9 cases of hematological complications in the LQ group and none in the HQ group. The breakdown of hematological diseases included 4 cases of monoclonal gammopathy of undetermined significance, 3 cases of myelodysplastic syndrome, 1 case of lymphoplasmacytic lymphoma, and 1 case of neutrophilic leukemia, all of which were untreated. There were no significant differences between the two groups in terms of gender, hemodialysis vintage, presence of diabetes, BNP, Kt/V, intravenous iron dosage, immunosuppressant medication, or hepatitis B vaccine response between the LQ and HQ groups. Serum albumin levels tended to be lower in the LQ group than in the HQ group, but the difference was not significant (*p* = 0.055).

**Table 2:** Comparison of dialysis patients in the lowest and highest antibody quartiles

	Lowest quartiles (LQ; *n* = 46) Anti-spike antibody IgG < 1,011 AU/mL	Highest quartiles (HQ; *n* = 46) Anti-spike antibody IgG > 7,688 AU/mL	*P*
Age	72.0 ± 10.2	65.4 ± 13.3	0.022
Male/Female	28/18	31/15	0.644
Dialysis vintage, months	121 ± 128	133 ± 98	0.988
DM/NDM	13/33	18/28	0.378
Serum albumin (g/dL)	3.61 ± 0.25	3.72 ± 0.24	0.055
Lymphocytes (/mm^3^)	940 ± 340	1219 ± 436	0.002
BNP (ng/mL)	328 ± 310	270 ± 317	0.188
Kt/V	1.42 ± 0.26	1.37 ± 0.31	0.637
PCR (g/kg)	45.4 ± 11.7	53.3 ± 18.8	0.097
nPCR (g/kg/day)	0.84 ± 0.19	0.94 ± 0.25	0.036
GNRI	91.7 ± 5.0	94.7 ± 5.1	0.004
Cr index (mg/kg/day)	86.2 ± 26.9	99.9 ± 28.0	0.024
BMI (kg/m^2^)	21.2 ± 3.8	22.9 ± 4.6	0.048
Intravenous iron dose (mg/6 months)	208 ± 194	205 ± 246	0.626
Current smoking (Yes/No)	11/35	3/40	0.041
Steroid (Yes/No)	9/37	1/45	0.015
Immunosuppressant (Yes/No)	1/45	0/46	1.000
Hematological disorder (Yes/No)	9/37	0/46	0.025
HBV vaccine responsiveness (Yes/No)	10/6	10/6	1.000

Values are means ± standard deviation

*DM* diabetes mellitus; *NDM* non-diabetes mellitus; *BNP* brain natriuretic peptide; *PCR* protein catabolic rate; *nPCR* normalized protein catabolic rate; *GNRI* geriatric nutritional risk index; *Cr* creatinine; *BMI* body mass index; and *HBV* hepatitis B virus

### Adverse reactions to BNT162b2 vaccine

**Fig. 2:**
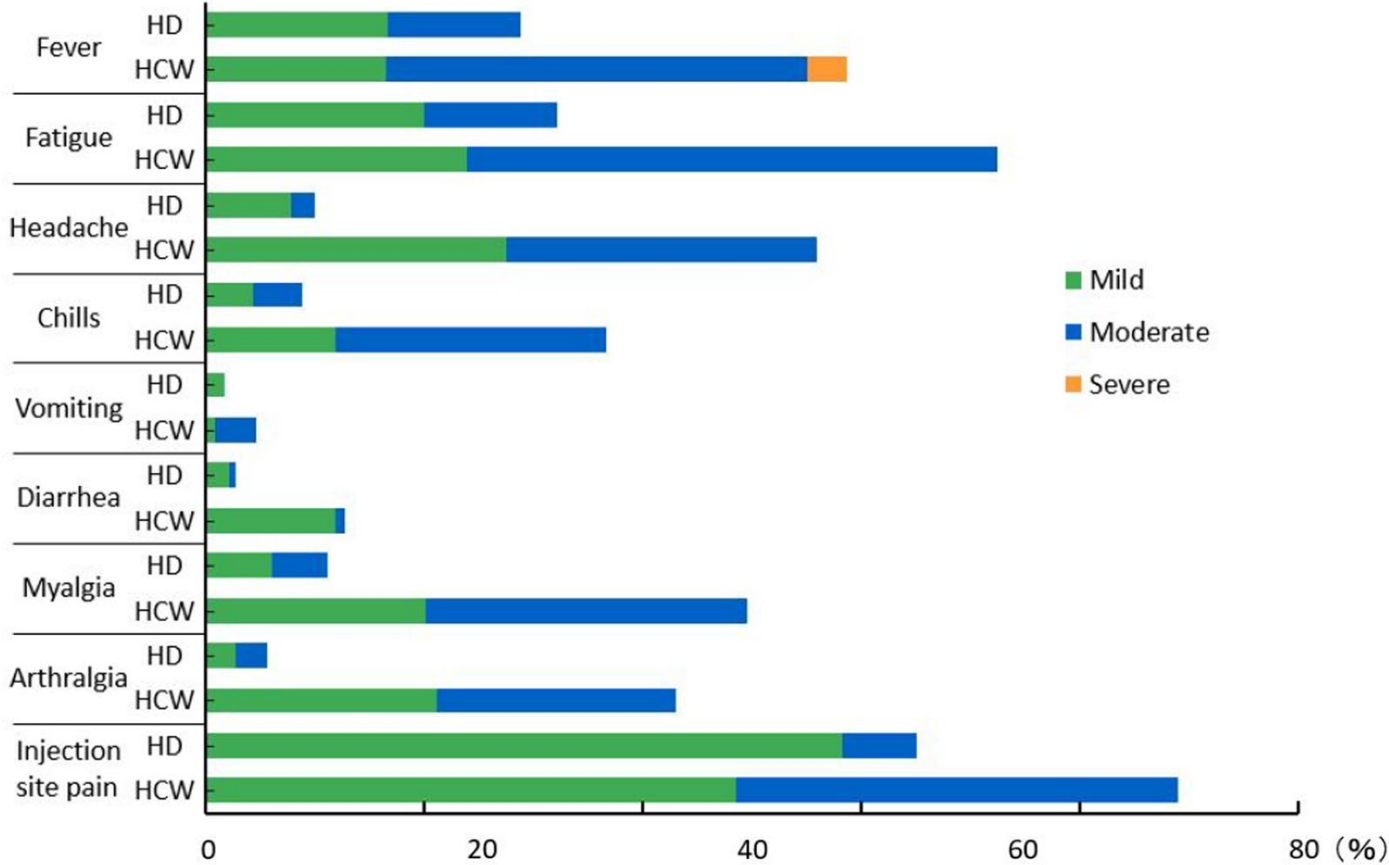
Adverse reactions to the BNT162b2 vaccine in the HD and HCW groups. With the exception of vomiting, adverse events were generally milder in the HD than the HCW group

Adverse events were generally milder in the HD group than in the HCW group, except for vomiting (Fig. 2), as previously reported [17, 18].

### Preventive effect of BNT162b2 vaccine on COVID-19 development

During the observation period, there were no cases of COVID-19 in the HCW group, whereas the HD group experienced two cases of COVID-19 breakthrough infection during this period. Both of those patients had already received two doses of the BTN162b2 vaccine but had not yet received the third dose. One patient had fever, sore throat, and a positive nasopharyngeal swab polymerase chain reactions test. The patient was treated with sotrovimab and molnupiravir at a public hospital and discharged after 10 days. The other patient was asymptomatic, but a cluster of COVID-19 occurred in the nursing home where the patient lived, and polymerase chain reactions tests of nasopharyngeal swabs were positive. The patient was asymptomatic and recovered with no special treatment.

## Discussion

Hemodialysis patients with COVID-19 are reportedly a higher risk of severe disease and mortality than the general population [2]. The best way to address this problem is to vaccinate against COVID-19 [3]. In our study, the vaccine positivity rate after two doses of BNT162b2 vaccine was 97.8% in the HD group and 100% in the HCW group. However, the median antibody titer in the HD group was significantly lower than in the HCW group (Fig. 1). There have been several earlier reports and review articles [4–16] on the effectiveness of COVID-19 vaccination in dialysis patients. According to a review from Carr et al. [6], the antibody acquisition rate after two doses of mRNA vaccine against COVID-19 in hemodialysis patients is 70–96%. In the present study, the factors associated with decreased fluid response after vaccination were older age, lower lymphocyte count, lower nPCR, lower GNRI, lower Cr index, lower BMI, steroid administration, and hematological disease. Age was found to be an independent predictor in some studies [8, 13–16] but not in others [26, 27]. As reported by Grupper et al. [19], a low lymphocyte count, low nPCR, and low GNRI, which are indicators of malnutrition, were factors in the low response to the vaccine. Kuwae et al. [28] reported that low lymphocyte counts in hemodialysis patients were associated with mortality and hospitalization rates, which are, in turn, associated with poor nutrition. In the present study, PCR also tended to be lower in weak responders than in stronger responders, but the difference was not significant. nPCR significantly differed between the HD and HCW groups, but PCR did not. We also observed that a low Cr index and low BMI were factors in the low vaccine response. Agur et al. [16] reported that hemodialysis patients with a BMI less than 30 responded well after administration of mRNA SARS-CoV-2 vaccine. By contrast, we found that patients with a lower BMI had a poorer response to the vaccine. This difference could be attributable to the difference in body sizes between Western and Japanese patients, as the mean BMI reported by Agur et al. [16] was 26.69 ± 5.51, which is larger than that in our HD group (22.2 ± 4.0). Steroid administration was more common in the LQ group, which is consistent with earlier reports [29, 30]. In the present study, it was not possible to assess the impact of immunosuppressive drugs on vaccine reactivity due to the small number of patients being administered them. Nine of the 46 patients in the LQ group experienced hematological complications as compared to none in the HQ group, which is a significant difference. The lymphocyte counts in the nine affected patients ranged from 642 to 1,693/mm3 (992 ± 312 /mm3), and many patients in the HD group had low lymphocyte counts, which may have contributed to the low vaccine response. On the other hand, two of the patients showing a weak response had lymphocyte counts of 1,300/mm3 or higher. Further studies with a larger number of patients will be needed to better understand the relation between lymphocyte counts and vaccine responses. Karoui et al. [7] reported that in multivariate analyses, use of immunosuppressive drugs, low serum albumin, low lymphocyte count, low IgG levels, hepatitis B vaccine non-responder status, high dialysis vintage, and high intravenous iron dosage were all independent predictors of a poor serological response. In our study, there was also a trend toward lower serum albumin levels in the weak responders, but the difference was not statistically significant (*p* = 0.055). Toda et al. reported that smoking history is associated with post-vaccine low antibody titers in hemodialysis patients [8]. However, we found that current smoking history, not smoking history, was associated with low antibody titer in our HD group. We could not assess reactivity to the hepatitis B vaccine due to the small number of vaccinated individuals overall. Patients in both groups had a long history of dialysis, and there was no significant difference in their histories of dialysis. The dose of intravenous iron in both groups was smaller than in other countries [31] and did not statistically differ. As previously reported by Simon [17] and Polewska [18], adverse events due to COVID-19 mRNA vaccination were milder in the HD group than the control group. This was thought to be due to the reduced immune response in the HD group. During the observation period, two COVID-19 breakthrough infections were observed in the HD group. In our region, about 93% of recent COVID-19 cases were caused by the Omicron variant. Given that the two breakthrough cases were mild or asymptomatic and had a good course, we suggest they were likely caused by the Omicron variant [32]. Because the anti-SARS-CoV-2 IgG antibody titer was lower in the HD than the HCW group, it is recommended that a third vaccination dose be administered as early as possible [33, 34].

## Conclusions

In conclusion, our findings indicate that the anti-SARS-CoV-2 IgG antibody titer was significantly lower in hemodialysis patients than in a healthy control sample after administration of two doses of the BNT162b2 vaccine. Factors associated with the low anti-SARS-CoV-2 IgG antibody titer in hemodialysis patients include old age, low lymphocyte count, low nPCR, low GNRI, low BMI, steroid use, and hematological disease.

## Data Availability

We wish to share our data.

## Abbreviations

AU: Arbitrary units
BMI: Body mass index
BNP: Brain natriuretic peptide
CI: Confidence interval
COVID-19: Coronavirus disease 2019
Cr: Creatinine
DM: Diabetes mellitus
GNRI: Geriatric nutritional risk index
HBV: Hepatitis B virus
HCW: Health care workers
HD: Hemodialysis
HQ: Highest quartile antibody titer
IgG: Immunoglobulin G
IQR: Interquartile range
LQ: Lowest quartile antibody titer
mRNA: Messenger ribonucleic acid
nPCR: Normalized protein catabolic rate
PCR: Protein catabolic rate
SARS-CoV-2: Severe acute respiratory syndrome coronavirus-2
SD: Standard deviation
